# Understanding the Correlation between Blood Profile and the Duration of Hospitalization in Pediatric Bronchopneumonia Patients: A Cross-Sectional Original Article

**DOI:** 10.2478/jccm-2024-0031

**Published:** 2024-07-31

**Authors:** Dessika Listiarini, Dev Desai, Yanuar Wahyu Hidayat, Kevin Alvaro Handoko

**Affiliations:** Dr. Soeselo General Hospital, Slawi, Central Java, Indonesia; Smt. NHL Municipal Medical College, Ahmedabad, Gujarat, India; Bhakti Rahayu Tabanan Hospital, Tabanan, Bali, Indonesia

**Keywords:** pediatric bronchopneumonia, complete blood count, pneumonia, duration of hospitalization, neutrophil-lymphocyte ratio, leukocyte, thrombocyte, length of stay

## Abstract

**Introduction:**

Pediatric bronchopneumonia is a prevalent life-threatening disease, particularly in developing countries. Affordable and accessible blood biomarkers are needed to predict disease severity which can be based on the Duration of Hospitalization (DOH).

**Aim of the Study:**

To assess the significance and correlation between differential blood profiles, especially the Neutrophil-Lymphocyte Ratio (NLR), and the DOH in bronchopneumonia children.

**Material and Methods:**

A record-based study was conducted at a secondary care hospital in Indonesia. After due ethical permission, following inclusion and exclusion criteria, 284 children with confirmed diagnoses of bronchopneumonia were included in the study. Blood cell counts and ratios were assessed with the DOH as the main criterion of severity. Mann-Whitney test and correlation coefficient were used to draw an analysis.

**Results:**

Study samples were grouped into DOH of ≤ 4 days and > 4 days, focusing on NLR values, neutrophils, lymphocytes, and leukocytes. The NLR median was higher (3.98) in patients hospitalized over 4 days (P<0.0001). Lymphocyte medians were significantly higher in the opposite group (P<0.0001). Thrombocyte medians were similar in both groups (P=0.44481). The overall NLR and DOH were weakly positively correlated, with a moderate positive correlation in total neutrophils and DOH, and a moderate negative correlation in total lymphocytes and DOH. The correlation between the DOH ≤ 4 days group with each biomarker was stronger, except for leukocyte and thrombocyte. Analysis of the longer DOH group did not yield enough correlation across all blood counts.

**Conclusions:**

Admission levels of leukocyte count, neutrophil, lymphocyte, and NLR significantly correlate with the DOH, with NLR predicting severity and positively correlated with the DOH.

## Introduction

Bronchopneumonia, the most common form of pneumonia in children, is suppurative inflammation in the lungs localized in both alveoli and forms patches around the bronchi [1]. According to WHO, it is the leading death-causing infectious disease in children globally, accounting for 14% of mortality in children < 5 years old and 22% in children aged 1 – 5 years [2]. In low-income and middle-income countries (LMICs) especially, pneumonia-related death creates a major burden of disease as well as healthcare-associated costs [4]. WHO recommends hospitalization only for severe cases of pneumonia [5]. Annually, 7% to 13% of approximately 156 million pneumonia cases worldwide require hospitalization. In LMICs, hospitalization average daily rate costs more than the average daily wage, taking 46.8% of a household’s total expenditure [6]. Often, this financial burden leads to “discharge against medical advice” (DAMA), creating more risks of readmission, complications, and even mortality in children [6].

Considering the continuously increasing cases and the drawbacks of hospital care, an affordable predictive biomarker is needed. Numerous studies have found that the Neutrophil-Lymphocyte Ratio (NLR) can be utilized as an inflammatory biomarker to predict prognosis and mortality in various cases [7,8,9,10]. It is also easily calculated from the differential white cell count [7]. In pneumonia, pathogen invasion of the lower respiratory tract results in inflammatory conditions starting from the site of infection [4]. However, there are very little studies about the NLR and other differential blood counts in relation to pneumonia outcomes in children. This outcome is measured by the duration of hospitalization (DOH) in this study. Understanding how long it takes from admission to discharge, is important for improving healthcare services quality. Therefore, this study aimed to evaluate the significance and correlation between differential blood profiles, especially the NLR, to the duration of hospitalization (DOH) among children with bronchopneumonia. Our study hypothesizes that the NLR has a significant positive correlation with the DOH.

## Materials And Methods

A record-based cross-sectional study was conducted at a secondary care hospital in Central Java, Indonesia. Pediatric patients with bronchopneumonia were included in the study to compare the blood cell levels to the severity of the disease characterized by the Duration of Hospitalization (DOH) of the patient. The diagnosis was confirmed using radiological proof along with clinical suspicion. The records from 1st January 2023 to 30th September 2023 were accessed after proper Ethics committee and Institutional Review Board permission and values like Neutrophil count, Leukocyte count, Lymphocyte count, and Thrombocyte count were recorded along with the length of stay (DOH) of the patients in the hospital. All comorbidities of the patients were also recorded along with other demographical records.

Inclusion Criteria:

- Pediatric patients whose complete set of records were available
- Pediatric patients with a definitive diagnosis of bronchopneumonia (clinical diagnosis supported by a positive chest radiograph)

Exclusion Criteria:

- Pediatric bronchopneumonia patients with congenital diseases
- Pediatric bronchopneumonia patients with comorbidities, including chronic diseases, malignancies, or malnutrition
- Patients taking other medications unrelated to bronchopneumonia
- Pediatric bronchopneumonia patients with other conditions that need any kind of surgery prolonging the DOH
- Patients who were transferred from or had been treated in another hospital
- Patients discharged against medical advice (DAMA) or being referred to higher-level centre
- Patients with incomplete data

NLR was counted using the absolute count from the records. The data were then assessed and analyzed using Correlation Coefficients and the Mann-Whitney test was used to find significant differences and draw correlations to conclude the results.

## Results

### Baseline Patients Characteristics

The baseline characteristics of the patients are shown in *Table 1*. In total, 284 patients fulfilled the study inclusion and exclusion criteria. Each group, DOH ≤ 4 days and DOH > 4 days, comprised 105 and 179 patients, respectively. Data collected were tested using the Kolmogorov-Smirnov test, and it showed abnormal distribution of data (p<0.05) across all variables (NLR, Lymphocyte, Neutrophil, Lymphocyte, and Thrombocyte).

**Table 1.** Baseline Patient Characteristics

**Characteristic**	**DOH ≤ 4 days (n=105)**	**DOH > 4 days (n=179)**	**Total (n= 284)**
Age (%)			
1 month - 1 year	33 (31.4%)	39 (21.8%)	72 (25.4%)
> 1 year	72 (68.6%)	140 (78.2%)	212 (74.6%)

Sex (%)			
Female	42 (40.0%)	86 (48.0%)	128 (45.1%)
Male	63 (60.0%)	93 (52.0%)	156 (54.9%)

IV Antibiotics (%)			
Ceftriaxone	44 (41.9%)	82 (45.8%)	126 (44.4%)
Ceftriaxone + Gentamicin	43 (41.0%)	68 (38.0%)	111 (39.1%)
Cefotaxime	7 (6.7%)	9 (5.0%)	16 (5.6%)
Cefotaxime + Gentamicin	7 (6.7%)	13 (7.3%)	20 (7.0%)
Ceftazidime	4 (3.8%)	7 (3.9%)	11 (3.9%)

DOH: Duration of Hospitalization; IV: Intravenous

The samples were divided into two age groups (1 month to 1 year and > 1 year), with the majority of the samples being > 1 year old (74.6%). The most used intravenous antibiotic was Ceftriaxone (44.4%), followed closely by a combination of Ceftriaxone and Gentamicin (39.1%). Cefotaxime in combination with Gentamicin was used in 7.0% of the samples, while Cefotaxime and Ceftazidime were used in 5.6% and 3.9%, respectively.

### Comparison of Differential Blood Profiles to DOH

There were significant differences between the two groups (≤ 4 days and > 4 days group) in terms of NLR value (p<0.0001), neutrophil (p<0.0001), lymphocyte (p<0.0001), and leukocyte (p=0.00039) (*Table 2*). However, there was no significant difference related to thrombocyte count (p=0.4448). The median value of the total NLR was 2.43, with a range of 0.11 to 73.20. Between the two groups, more patients were hospitalized for > 4 days. The NLR, neutrophil, and leukocyte medians were higher in patients hospitalized for more than 4 days. However, the median of lymphocytes was significantly higher in the opposite group (≤ 4 days) with 46.4. The shorter (≤ 4 days) and longer (> 4 days) DOH groups, however, yielded similar thrombocytes median of 338 and 353, respectively.

**Table 2.** Statistical Analysis: Significance and Correlation between the Differential White Cell Counts and DOH

**Differential Blood Profiles (on the day of admission)**	**Reference Range**	**Parameter**		**Median (Minimum-Maximum)**	**P-value**	**Correlation Coefficient (R)**
NLR	< 3.13	DOH	≤ 4 days	0.89 (0.11–1.84)	<0.0001*	0.6495
			> 4 days	3.98 (0.21–73.20)		0.2439
		Total		2.43 (0.11–73.20)		0.3206

Neutrophils (%)	50–70	DOH	≤ 4 days	41.7 (4.14–60.8)	<0.0001*	0.7363
			> 4 days	72 (15–92.2)		−0.1828
		Total		62.9 (4.14–92.2)		0.4396

Lymphocytes (%)	25–40	DOH	≤ 4 days	46.4 (6.68–81.5)	<0.0001*	−0.7713
			> 4 days	18.5 (2.54–70.2)		0.1095
		Total		26.75 (2.54–81.5)		−0.4889

Leukocytes (10^9^/L)	5.0–21.0	DOH	≤ 4 days	10.2 (2.1–32.3)	0.00039*	−0.0758
			> 4 days	12.9 (3.0–54.6)		0.0393
		Total		11.95 (2.1–54.6)		0.1523

Thrombocytes (10^9^/L)	150–400	DOH	≤ 4 days	338 (93–849)	0.44481	−0.0714
			> 4 days	353 (22.6–732)		−0.0688
		Total		346 (22.6–849)		−0.0299

* Mann-Whitney test is significant at p-value < 0.05; DOH: Duration of Hospitalization; NLR: Neutrophil/Lymphocyte Ratio

There was a weak positive correlation (R=0.321) between the overall NLR value and the DOH, and between the leukocytes and the DOH (R= 0.1523). In addition, a stronger, moderate positive correlation (R=0.4396) was found between the total neutrophil and the DOH. On the other hand, there was also a moderate negative correlation (R= −0.4889) between the total lymphocytes and DOH. Analysis of only the DOH ≤ 4 days group, however, showed that both NLR and neutrophil had strong positive correlations with the DOH, while lymphocytes had a strong negative correlation with the DOH. Meanwhile, leukocytes and thrombocytes in this group showed almost no correlation to the DOH. Analysis of the longer DOH group did not yield any strong enough correlation to the DOH across all differential blood profiles.

## Discussions

In this study, there were more bronchopneumonia children above one year old compared to those in the age range of 1 month to 1-year-old, with more males than females. These findings were similar to a recent case-control study of childhood pneumonia in high-prevalence areas of Indonesia that reported the biggest proportion of children was in the age range of 1 – 3 years (51.14%), but with more females (51.14%) than males [11]. Another study also found more pneumonia patients in the age range of 12–59 months (61.3%), as well as more males than females (58.1%) [12]. On the contrary, two studies conducted in Bali reported more pneumonia patients aged less than one year old [12, 13]. Pneumonia is easier to contract in children < 5 years old since their immunity has not fully matured and their airways are relatively narrow, increasing the risk of secretion blockage and bacterial pathogen colonization [14]. Male sex was also reported as one of the pneumonia risk factors due to the airway diameters being smaller in male children. Some differences in immunology were also found to be related to sex [15].

The most used intravenous (IV) antibiotic in our patients was Ceftriaxone, followed by the joint use of Ceftriaxone and Gentamicin. Cefotaxime, combined with gentamicin or as a single regimen, and Ceftazidime were also used in smaller proportions of the patients. The regimen and its route of administration follow several guidelines. According to WHO, children with tachypnea should be considered as pneumonia and treated with take-home antibiotics. However, those with chest retractions or any danger signs indicating severe pneumonia are advised to receive in-hospital treatment, particularly IV antibiotics [16]. Indonesian Pediatric Society Medical Service Guidelines 2009 recommends several choices of antibiotic regimens including ampicillin and chloramphenicol, co-amoxiclav, ceftriaxone, cefuroxime, and cefotaxime. In children < 2 months old, a combination of ampicillin and gentamicin is highly recommended [17]. Rational use of antibiotics in Community Acquired Pneumonia (CAP) is important to prevent longer DOH, incomplete treatment, and increased antibiotic resistance [18, 19].

In our study, NLR, measured at admission, showed a significant difference between the DOH ≤ 4 day group and the DOH > 4 days group (p<0.0001). We also found a higher median of 3.98 in the longer (> 4 days) DOH group. Rising levels of NLR insinuate an active inflammatory immune response to various pneumonia pathogens infection. Several studies have mentioned this activation and dysregulation of the immune system as the cause of neutrophils and lymphocyte component changes [20, 21]. In response to proinflammatory cytokines and chemokines, neutrophils will be recruited to the site of infection. In severe cases, immature neutrophils will also be continuously released from the bone marrow. Meanwhile, several anti-inflammatory cytokines cause immunosuppression and apoptosis in many lymphocytes. This leads to neutrophilia and lymphocytopenia, which then elevate the NLR. As an inflammatory biomarker, an increased NLR indicates a more severe form of infection, which can cause a prolonged DOH [20, 22].

Previous studies of NLR in other pediatric cases reported similar results. One study by Puratmaja et al. investigated the correlation between NLR and bacterial infection as indicated by Procalcitonin (PCT) and C-Reactive Protein (CRP) levels in chronic kidney disease children. They found a significant positive correlation between NLR with PCT levels but not with CRP levels. Even so, this could be caused by sample size difference, and they concluded that NLR could possibly be used as an alternative bacterial infection marker [23]. Their results align with ours. Another study by Lutfi et al. reported a higher risk and more severe form of infection with findings of bilateral pneumonia in Covid-19 children who had NLR > 3.3 [24]. A cohort study investigating the use of NLR as one of the biomarkers to differentiate childhood pneumonia from upper respiratory tract infection (URTI) also reported a superior discriminative accuracy of NLR compared to other biomarkers. However, some results might be in contrast to our study as NLR was reported to decrease the risks of pneumonia while lymphocyte-to-monocyte ratio (LMR) increased the risks [25]. Although LMR was not assessed in this study, a significantly higher lymphocytes median was observed in the shorter DOH group. A study by Zheng et al. assessing the NLR role in diagnosing bacterial childhood pneumonia also reported a significantly higher value as well as a positive correlation between NLR and serum IL-6 [26]. IL-6 is a pleiotropic cytokine secreted upon infection, and elevation is usually seen in *S. pneumonia* infection [27]. Although bacterial culture from samples is not routinely performed in a resource-limited setting and was not evaluated within this study, in line with our hypothesis, we found a significantly higher NLR median in the longer DOH group.

Positively correlated (R=0.3206), a higher NLR level indicates a longer hospitalization duration that could signify a more severe form of childhood bronchopneumonia requiring longer treatment and recovery time. NLR has been used to predict high risk of prolonged DOH and poor prognosis of different cases in the pediatric population. A study by Miguel et al. in the pediatric population showed that NLR at the time of first diagnosis of acute appendicitis was strongly and positively correlated with the DOH post-appendectomy. This study suggested the use of NLR to predict a high risk of prolonged DOH in appendicitis children [28]. NLR was also able to predict worse outcomes in the postoperative period of heart surgery in children. A higher NLR was reported to have a significant association with extended DOH in the hospital and ICU [29]. In one study by Arwas et al., children with higher NLR numbers were found to have a more severe clinical course of asthma. They also revealed a significant correlation between the NLR and the need to be treated in the resuscitation room, indicating the requirement for more intensive care for these children [30]. Although very few studies are available evaluating the correlation between NLR and the DOH in the bronchopneumonia pediatric population, we found one similar study but with a much smaller number of samples at one of Indonesia’s local hospitals. Their study revealed a significantly higher median NLR value in pneumonia children with longer DOH, although the correlation was not assessed [31]. The result of the present study is essentially consistent with those of the previously mentioned studies in terms of higher NLR levels indicating a worse clinical manifestation that requires prolonged treatment, which is reflected by the longer DOH.

Related to the leukocyte count, this study found a significant difference between the two DOH groups and a positive correlation, indicating that the higher the leukocyte count, the longer the DOH of bronchopneumonia children. Leukocyte count has been used by practitioners to aid the diagnosis of pneumonia, to measure its severity, and to predict the outcome, including the DOH. It was reported that patients with bacterial pneumonia have a leukocyte count of more than 15,000/mm^3^, even though in 5–10% of cases, leukocyte count can be lower than 6,000/mm. A study in 2015 showed that the majority of hospitalized pneumonia children had leukocytosis (leukocyte count >10,000/mm^3^). However, several studies reported some limitations regarding the predictive ability of leukocyte count related to disease outcomes [32, 33].

Our finding showed no significant difference in the median thrombocyte count between the two DOH groups. However, a weak negative correlation was observed, suggesting that the lower the level of thrombocyte count, the longer the DOH would be. These results align with studies that have reported thrombocytopenia predicts disease severity and adverse outcomes through an increased PSI or CURB65 score [34,35,36,37,38]. Changes in platelet levels significantly increased the rate of readmission, 30-day mortality, and longer DOH in CAP patients compared to those with normal counts [34, 35]. In acute infections, a common falling level of thrombocytes can be due to several reasons, such as declined production, increased cell death, and/or platelet aggregation [39, 40]. Thrombocytes, upon interacting with activated neutrophils via their Toll-like Receptors 4 (TLR4), form neutrophil extracellular traps (NET), which in turn amplify platelet activation and aggregation [41]. Alternatively, rising thrombocytes indicate a compensatory mechanism for infection and inflammation by enhancing megakaryocyte production in the bone marrow [40].

Possible limitations to this study are attributed to the fact that it was a retrospective single-centre study that might contribute to sample selection bias. However, we have minimised this by defining our research hypothesis and excluding DAMA and referral cases. Further larger, prospective, and multi-centred studies are still needed to gather more evidence, confirming the predictive value of the NLR and other blood differentials in paediatric bronchopneumonia cases.

## Conclusion

From our results, we can conclude that initial admission levels of NLR hold a prognostic value to predict the severity of pediatric bronchopneumonia, reflected by the duration of hospitalization (DOH). The NLR is a promising alternative biomarker since it is widely accessible in developing countries and easily obtained early into patients’ admission. In addition, leukocyte count, neutrophils, and lymphocytes also showed significant differences, useful to determine the DOH.
